# P03-11 Talking to Danish children/adolescents living with cerebral palsy and their parents about perceived motivators and barriers for being physically active - a family perspective

**DOI:** 10.1093/eurpub/ckac095.047

**Published:** 2022-08-29

**Authors:** Sofie Morley, Jens Troelsen, Anders Holsgaard Larsen, Christina Esmann Fonvig

**Affiliations:** 1 Department of Sports Science and Clinical Biomechanics, University of Southern Denmark, Odense, Denmark; 2 The Orthopaedic Research Unit, Department of Clinical Research, University of Southern Denmark, Department of Orthopaedics and Traumatology, Odense University Hospital, Odense, Denmark

**Keywords:** cerebral palsy, physical activity, family, perceived motivation and barriers

## Abstract

**Background:**

Children and adolescents with cerebral palsy (CP) are a vulnerable group who find it challenging to meet current physical activity guidelines, which predispose them to the negative health implications associated with low levels of physical activity and high levels of sedentary time. For these reasons, a key role for many clinicians, parents, and other practitioners working with children and adolescents with cerebral palsy is to encourage and facilitate an increase in habitual physical activity and reduce the amount of time spent sedentary, in order to optimize long-term health outcomes. Since 2014 Danish schools have been committed to enhance physical activity during the school day, but teachers still find it challenging to include children and adolescents with special needs. In Denmark, there is a strong tradition of practicing habitual exercise in the voluntary sports clubs (83% of children and adolescents). In sports clubs, these children are being physically active as well as experiencing being a part of a community. Children and adolescents living with CP are often not able to participate in these sports clubs which excludes them from the active and social life that's happening there. This study acknowledges that parents of children and adolescents living with CP play an important role in supporting them being physically active. Therefore, this study aims to identify perceived barriers and motivators for being physically active, experienced by this particular group of children and parents. This knowledge can be used by parents, clinicians, coaches, teachers and other practitioners to guide families living with CP towards a more physically active lifestyle and possibly optimize long-term physical and social health outcomes for children and adolescents with CP.

**Methods:**

This study will investigate the children's and parents' perceptions of motivators and barriers. The study is designed as a multi-family member interview study involving 10-14 combined in-depth interviews with children aged 8-15 (GMFCS I-III) and their parents. Interviews will be analysed thematically within and between groups.

**Results and conclusions:**

The study will take place in spring 2020 as a part of a pre-graduate research year and thus data and conclusions will be presented at the conference.

